# Evaluation of crystal violet decolorization assay for minimal inhibitory concentration detection of primary antituberculosis drugs against *Mycobacterium tuberculosis* isolates*

**DOI:** 10.1590/0074-02760160082

**Published:** 2016-06-10

**Authors:** Ahmet Yilmaz Coban, Ahmet Ugur Akbal, Meltem Uzun, Yeliz Tanriverdi Cayci, Asuman Birinci, Belma Durupinar

**Affiliations:** 1 Ondokuz Mayis University Medical School, Department of Medical Microbiology, Samsun, Turkey; 2 Istanbul University Istanbul Medical School, Department of Medical Microbiology, Istanbul, Turkey

**Keywords:** Mycobacterium tuberculosis, antituberculosis drugs, susceptibility testing, multi drug resistance, crystal violet decolorization assay

## Abstract

In this study we evaluated the crystal violet decolorization assay (CVDA) for detection of minimum inhibitory concentration (MIC) of antituberculosis drugs. 53 isolates were tested in this study and 13 of them were multidrug resistant (MDR) isolates. The antibiotics concentrations were 2-0.06 mg/L for isoniazid (INH) and rifampicin (RIF) and were 16-0.25 mg/L for streptomycin (STM) and ethambutol (EMB). Crystal violet (CV-25 mg/L) was added into the microwells on the seventh day of incubation and incubation was continued until decolorization. Decolorization of CV was the predictor of bacterial growth. Overall agreements for four drugs were detected as 98.1%, and the average time was detected as 9.5 ± 0.89 day after inoculation. One isolate for INH and two isolates for STM were determined resistant in the reference method, but susceptible by the CVDA. One isolate was susceptible to EMB by the reference method, but resistant by the CVDA. All results were concordant for RIF. This study shows that CVDA is a rapid, reliable and suitable for determination of MIC values of *Mycobacterium tuberculosis*. And it can be used easily especially in countries with limited-sources.

Tuberculosis (TB) remains one of the world’s deadliest communicable diseases. In 2013, an estimated 9.0 million people developed TB and 1.5 million died from the disease. The proportion of new cases with multidrug-resistant TB (MDR-TB) was 3.5% in 2013 and has not changed compared with recent years, in the worldwide (WHO 2014). Early and accurate detection of drug resistance in TB, especially MDR and extensively drug-resistance (XDR) is the most important step for the use of appropriate treatment regimens for the patient, which has an important impact for the better control of the disease (Martin et al. 2008, Coban et al. 2014a). The development of rapid methods for drug susceptibility testing (DST) is very important due to the increasing rates of MDR-TB and the recently described XDR-TB in the worldwide (Aziz et al. 2006, Shah et al. 2007).

It is well known that proportion method is gold standard for detection of drug resistance in TB. However, obtaining results require three-six weeks. This problem can be dissolved by the use of automated systems such as Bactec MGIT 960 (Becton Dickinson Diagnostic Systems, Sparks, MD, USA), but it has high cost and need for equipment (CLSI 2011). For these reasons, their use of developing countries is limited. In addition, molecular methods of susceptibility testing are available, including the expensive commercial Xpert MTB/RIF and Genotype MTBDRplusassays (Bwanga et al. 2009, Friedrich et al. 2011, Chang et al. 2012).

Recently, new rapid, inexpensive, reliable and reproducible colorimetric methods including nitrate reductase assay (NRA), resazurin microtiter assay (REMA), malachite green decolorization assay and crystal violet decolorization assay (CVDA) have been developed (Farnia et al. 2008, Coban & Uzun 2013, Coban 2014, Coban et al. 2014a, b).

In this study, CVDA was evaluated to detect minimum inhibitory concentration (MIC) for primary antituberculosis drugs against *Mycobacterium tuberculosis*.

## MATERIALS AND METHODS

*Bacterial isolates* - In this study, 53 isolates obtained from pulmonary tuberculosis patients were used. The isolates that tested in this study were different from other studies. 13 isolates were MDR, 14 isolates were only resistant to isoniazid (INH) and one isolate was only resistant to rifampicin (RIF). Remaining 25 isolates were susceptible to both of INH and RIF. 22 isolates were resistant to streptomycin (STM) and 12 isolates were resistant to ethambutol (EMB). In addition, H37Rv (susceptible to all drugs), ATCC 35822 (resistant to INH), ATCC 35838 (resistant to RIF), ATCC 35837 (resistant to EMB) and ATCC 35820 (resistant to STM) were used as control isolates. All isolates were sub-cultured on LJ medium.

*Preparation of the medium* - Middlebrook 7H9S broth (containing 0.1% casiton, 0.5% glycerol and 10% oleic acid, albumin, dextrose and catalase-OADC) was used in the study. For preparation of inoculums, Middlebrook 7H9 broth without casiton and OADC was used.

*Preparation of antibiotics and crystal violet (CV)* - Stock solution of CV was prepared at 25 mg/L with sterile distilled water. This suspension of CV was sterilised by filtration and stored at 4ºC until use. Stock solutions were prepared at 1000 mg/L for STM, INH, RIF and EMB. Methanol was used as solvent for RIF, and sterile distilled water was used for others. The stock solutions of antibiotics were stored at -40ºC until use.

*Preparation of test microplates* - All tests were performed in 96-well microtitre plates. All wells were filled with 0.1 mL of Middlebrook 7H9S broth. Antibiotic test concentrations were prepared by the serial two-fold dilution. Seven dilutions of each antibiotics and a growth control well prepared for each isolates. The antibiotics concentrations were 2-0.06 mg/L for INH and RIF and were 16-0.25 mg/L for STM and EMB. All prepared microtitre plates were stored at -40ºC until use.

*Preparation of bacterial inoculum* - Freshly sub-cultured bacterial isolates were used for preparation of inoculum. All colonies of each isolates from LJ media were transferred into tubes containing 5 mL Middlebrook 7H9 broth without casiton and OADC and 15-20 glass beads and were vortexed for 1-2 min. The tubes were kept in vertical position for 30 min at room temperature to allow to precipitation of aerosol and other particles. The turbidity of the supernatant was adjusted to a McFarland standard number 1. The prepared bacterial suspension was then diluted at an 1:10 ratio in Middlebrook 7H9S broth.

*Application of the test* - 100 microlitres of bacterial suspension was inoculated into each well of the plates. After bacterial inoculation, all plates were incubated at 37ºC under normal atmospheric conditions. On the seventh day of incubation, 25 μL of CV (25 mg/L) were added into the growth control and drug containing wells simultaneously. After that incubation was continued until decolorized of CV in the growth control well. MIC was defined as the lowest drug concentration without decolorization (Figure). If the MIC value was over the breakpoints value, isolate was considered to be resistant to that tested antibiotics. Breakpoints values were 0.125, 0.5, 2 and 4 mg/L for INH, RIF, STM and EMB, respectively.

## RESULTS

In this study, resistance patterns and MIC values of standard strains were summarised in Table I. MIC values of H37Rv were 0.06, 0.06, 0.5 and 1 mg/L for INH, RIF, STM and EMB, respectively. MIC values of ATCC 35822 (INH resistant) were > 2, 0.06, 1 and 2 mg/L for INH, RIF, STM and EMB, respectively. MIC values of ATCC 35838 (RIF resistant) were 0.06, > 2, 0.5 and 2 mg/L for INH, RIF, STM and EMB, respectively. MIC values of ATCC 35820 (STM resistant) were < 0.03, 0.03, > 16 and < 0.25 mg/L for INH, RIF, STM and EMB, respectively. MIC values of ATCC 35837 (EMB resistant) were 0.125, 0.06, 1 and 16 mg/L for INH, RIF, STM and EMB, respectively. All results for standard strains were obtained between 9-14 days (Table I).

**TABLE I:** Minimal inhibitory concentration values of control strains

Isolates	Reference method*				CVDA (MIC value-mg/L)				Day
	
	INH	RIF	STM	EMB	INH	RIF	STM	EMB	
H37Rv	S	S	S	S	0.06 (S)	0.06 (S)	0.5 (S)	1 (S)	14
ATCC35822	R	S	S	S	> 2 (R)	0.06 (S)	1 (S)	2 (S)	10
ATCC35838	S	R	S	S	0.06 (S)	> 2 (R)	0.5 (S)	2 (S)	9
ATCC35820	S	S	R	S	< 0.03 (S)	0.03 (S)	> 16 (R)	< 0.25 (S)	13
ATCC35837	S	S	S	R	0.125 (S)	0.06 (S)	1 (S)	16 (R)	11

*Reference method: Bactec MGIT 960; CVDA: crystal violet decolorization assay; EMB: ethambutol; INH: isoniazid; R: resistant; RIF: rifampicin; S: susceptible; STM: streptomycin.

In the study, 24 isolates were susceptible all primary antituberculous agents. MIC values of INH and RIF were < 0.03 mg/L for 13 isolates and were 0.06 mg/L for 11 isolates. For STM, MIC values were < 0.03 mg/L for 13 isolates, 0.5 mg/L for seven isolates and 1 mg/L for four isolates. For EMB, MIC values were 1 mg/L for 10 isolates and 2 mg/L for 14 isolates. All results were obtained between eight-10 days (Table II).

**TABLE II:** Minimal inhibitory concentration values of drug susceptible isolates

Isolate No	Reference method*				CVDA (MIC value-mg/L)				Day
	
	INH	RIF	STM	EMB	INH	RIF	STM	EMB	
4	S	S	S	S	< 0.03/S	< 0.03/S	< 0.25/S	1/S	10
5	S	S	S	S	< 0.03/S	< 0.03/S	< 0.25/S	1/S	10
6	S	S	S	S	< 0.03/S	< 0.03/S	< 0.25/S	1/S	10
7	S	S	S	S	< 0.03/S	<0.03/S	<0.25/S	1/S	10
8	S	S	S	S	< 0.03/S	< 0.03/S	< 0.25/S	2/S	10
9	S	S	S	S	< 0.03/S	< 0.03/S	< 0.25/S	2/S	10
11	S	S	S	S	< 0.03/S	< 0.03/S	< 0.25/S	1/S	10
13	S	S	S	S	< 0.03/S	< 0.03/S	< 0.25/S	2/S	10
16	S	S	S	S	< 0.03/S	< 0.03/S	< 0.25/S	1/S	10
17	S	S	S	S	< 0.03/S	< 0.03/S	< 0.25/S	1/S	10
20	S	S	S	S	< 0.03/S	< 0.03/S	< 0.25/S	1/S	10
30	S	S	S	S	< 0.03/S	< 0.03/S	< 0.25/S	2/S	10
31	S	S	S	S	< 0.03/S	< 0.03/S	< 0.25/S	1/S	10
32	S	S	S	S	0.06/S	0.06/S	1/S	2/S	9
33	S	S	S	S	0.06/S	0.06/S	1/S	2/S	9
34	S	S	S	S	0.06/S	0.06/S	1/S	2/S	9
35	S	S	S	S	0.06/S	0.06/S	0.5/S	2/S	8
38	S	S	S	S	0.06/S	0.06/S	0.5/S	1/S	8
41	S	S	S	S	0.06/S	0.06/S	0.5/S	2/S	8
42	S	S	S	S	0.06/S	0.06/S	0.5/S	2/S	8
44	S	S	S	S	0.06/S	0.06/S	0.5/S	2/S	9
45	S	S	S	S	0.06/S	0.06/S	1/S	2/S	9
46	S	S	S	S	0.06/S	0.06/S	0.5/S	2/S	9
48	S	S	S	S	0.06/S	0.06/S	0.5/S	2/S	9

*Reference method: Bactec MGIT 960; CVDA: crystal violet decolorization assay; EMB: ethambutol; INH: isoniazid; R: resistant; RIF: rifampicin; S: susceptible; STM: streptomycin.

29 isolates, which have various resistant patterns, were tested in the study. 10 MDR isolates were resistant to INH, RIF, STM and EMB, and three MDR isolates were resistant to INH, RIF and STM. Nine isolates were resistant to INH and STM, five isolates were only resistant to INH, one isolate was resistant to RIF and one isolate was resistant to STM. All results were obtained between eight-12 days (Table III).

**TABLE III:** Minimal inhibitory concentration values of drug resistant isolates

Isolate No	Reference method*				CVDA (MIC-value-mg/L)				Day
	
	INH	RIF	STM	EMB	INH	RIF	STM	EMB	
1	R	R	R	R	> 2/R	> 2/R	8/R	8/R	10
2	R	R	R	R	> 2/R	> 2/R	> 16/R	> 16/R	10
3	R	S	R	S	2/R	< 0.03/S	> 16/R	< 0.25/S	10
10	R	R	R	R	> 2/R	> 2/R	4/R	8/R	10
12	R	S	S	S	0.25/R	< 0.03/S	< 0.25/S	1/S	10
14	R	S	R	S	0.25/R	< 0.03/S	4/R	1/S	10
15	R	S	S	S	0.25/R	< 0.03/S	< 0.25/S	1/S	10
18	R	R	R	S	2/R	> 2/R	> 16/R	2/S	10
19	R	R	R	R	> 2/R	> 2/R	> 16/R	16/R	10
21	R	R	R	R	> 2/R	> 2/R	16/R	8/R	10
22	R	S	R	S	0.25/R	0.06/S	> 16/R	2/S	10
23	***R***	S	***R***	S	***0.125/S***	< 0.03/S	***1/S***	2/S	10
24	R	S	S	S	0.25/R	< 0.03/S	< 0.25/S	1/S	10
25	R	S	R	S	2/R	0.125/S	8/R	2/S	10
26	S	S	R	S	< 0.03/S	0.25/S	> 16/R	2/S	10
27	R	S	R	S	0.25/R	< 0.03/S	4/R	1/S	10
28	R	S	S	S	0.25/R	< 0.03/S	< 0.25/S	< 0.25/S	10
29	R	R	R	R	> 2/R	> 2/R	> 16/R	8/R	10
36	R	R	R	R	2/R	> 2/R	8/R	16/R	9
37	R	R	R	***S***	2/R	> 2/R	16/R	***8/R***	8
39	R	S	***R***	S	0.5/R	0.25/S	***2/S***	2/S	8
40	R	S	R	S	2/R	0.25/S	> 16/R	2/S	8
43	R	R	R	S	> 2/R	> 2/R	> 16/R	4/S	12
47	R	R	R	R	> 2/R	> 2/R	> 16/R	16/R	8
49	R	S	S	S	2/R	0.125/S	0.5/S	2/S	11
50	S	R	S	S	0.06/S	> 2/R	0.5/S	1/S	10
51	R	S	R	S	> 2/R	0.25/S	4/R	4/S	9
52	R	R	R	R	> 2/R	> 2/R	> 16/R	8/R	8
53	R	R	R	R	> 2/R	> 2/R	> 16/R	8/R	8

*Reference method: Bactec MGIT 960; CVDA: crystal violet decolorization assay; EMB: ethambutol; INH: isoniazid; R: resistant; RIF: rifampicin; S: susceptible; STM: streptomycin.

The sensitivity, specificity, positive predictive value (PPV), negative predictive value (NPV) and agreement for INH were 96.3%, 100%, 100%, 96.3% and 98.1%, respectively. One isolate was resistant to INH by the reference method, but susceptible by the CVDA. All results were concordant for RIF and all values were 100%. The sensitivity, specificity, PPV, NPV and agreement for STM were 91.3%, 100%, 100%, 93.7% and 96.2%, respectively. Two isolates were resistant to STM by the reference method, but susceptible by the CVDA. The sensitivity, specificity, PPV, NPV and agreement for EMB were 100%, 97.6%, 90.9%, 100% and 98.1%, respectively. One isolate was susceptible to EMB by the reference method, but resistant by the CVDA (Table IV). Overall agreement for four drugs was detected as 98.1%, and the average time was detected as 9.5 ± 0.89 day after inoculation.

**TABLE IV:** Comparison of the result of crystal violet decolorization assay (CVDA) with those obtained with reference method

Drugs	CVDA	Reference method*		Sensitivity (%)	Specificity (%)	PPV (%)	NPV (%)	Agreement (%)

		R	S					
INH	R	26	0	96.3	100	100	96.3	98.1
	S	1	26					
RIF	R	14	0	100	100	100	100	100
	S	0	39					
STM	R	21	0	91.3	100	100	93.7	96.2
	S	2	30					
EMB	R	10	1	100	97.6	90.9	100	98.1
	S	0	42					

*Reference method: Bactec MGIT 960; EMB: ethambutol; INH: isoniazid; NPV: negative predictive value; PPV: positive predictive value; R: resistant; RIF: rifampicin; S: susceptible; STM: streptomycin.

**Figure:**
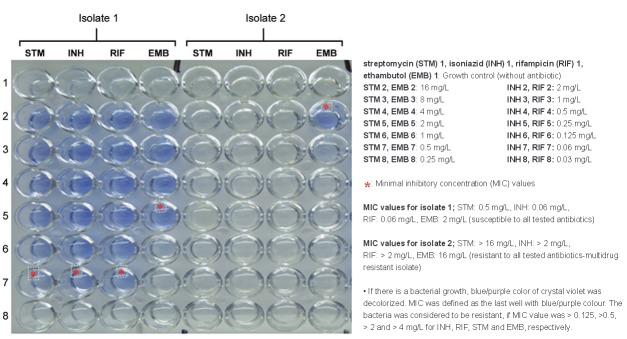
Evaluation of minimal inhibitory concentration plate.

## DISCUSSION

Early detection of tuberculosis, especially MDR-TB, allows the effective treatment of TB patients and contributes the TB control. Therefore rapid detection methods for susceptibility testing of *M. tuberculosis* are crucial. Several rapid, inexpensive, reliable and accurate colorimetric and phenotypic methods have been developing (WHO 2011). Colorimetric methods have some advantages such as they are rapid, accurate, reliable, easy perform, inexpensive and evaluate by the naked eye. The NRA and REMA are well known but malachite green decolorization assay and CVDA have been newly developed.

In the REMA, the pooled sensitivity for INH, RIF, EMB and STM was 96, 97, 92 and 92, respectively. Pooled specificity for INH, RIF, EMB and STM was 96, 99, 86 and 90, respectively. The results had been obtained in eight-nine days (Coban et al. 2014a). Nour et al. (2013) determined the sensitivities, specificities, PPVs, NPVs and agreements of INH and RIF were 100% by REMA.

In the NRA, the pooled sensitivities-specificities were 96-99% for INH, 97-100% for RIF, 90-98% for EMB, and 82-96% for STM. The results had been obtained between five and 28 days by the direct test and between five and 14 days by the indirect test (Coban et al. 2014b). Montoro et al. (2005) reported that the sensitivity and specificity of the NRA for INH and RIF were 95.6% and 100%, respectively.

It was reported that water-born pathogenic mycobacteria were resistant to CV and they decolorized CV. This feature is membrane associated and resistance could be due to the reduction of CV and the sequestering in the lipid fraction (Jones & Falkinham 2003). After this knowledge, CVDA for early detection of MDR-TB was developed by Coban in 2014. It was reported that agreements for INH and RIF were 94.5-98% and 96.3-98%, respectively (Coban 2014, Coban et al. 2015).

In this study, we determined the MIC values of four drugs by CVDA and agreements were 98.1%, 100%, 96.2% and 98.1% for INH, RIF, STM and EMB, respectively. Overall agreement for four drugs was detected as 98.1%, and the average time was detected as 9.5 ± 0.89 day after inoculation. Similar results as other colorimetric assay including the REMA and the NRA were also obtained by CVDA.

The issue for managers of TB laboratories, particularly in resource-limited settings, has been to interpret the biosafety levels into specific precautions relevant to a country’s activities. The probability of aerosols being generated is important for determining the level of risk. Using liquid medium has an increased risk of generating aerosols; thus, it is recommended to perform these procedures should be in a biosafety cabin (BSC) (WHO 2012). Also, BSC is important for the prevention of contaminations in TB testing. However, BSC and using of personal protective equipments for biosafety sometimes may not be cost effective in countries with limited resources.

In conclusion, CVDA is a rapid, cheap, reliable and suitable for determination of MIC values of *M. tuberculosis*. In addition, it can be used for screening of new antitubercular chemicals. Even if further multicenter studies are needed prior to use in routine laboratory, it is promised for used in developed and developing countries.
